# Exploring Glucagon‐Like Peptide‐1 Receptor Agonists Usage Among Non‐Diabetic Healthcare Providers: A Cross‐Sectional Multi‐Country Study

**DOI:** 10.1002/hsr2.70638

**Published:** 2025-04-23

**Authors:** Abdulqadir J. Nashwan, Hana J. Abukhadijah, Vidusha Karavadi, Ibrahim Aqtam, Anas Ibraheem, Prakash Palanivelu, Mahmoud A. Khedr, Abdulkarim O. Agga, Obaid Ur Rehman, Eeshal Fatima, Mohammad A. Abu Asal, Rana Abutaima, Marwa M. Shaban, Mostafa Shaban, Muna Barakat, Nasser M. Aldosari, Albara M. Alomari, Adham A. Aljariri, Nabeel F. Al‐Lobaney, Mutaz I. Othman, Ahmad A. Abujaber, Kholoud Bastaki

**Affiliations:** ^1^ Department of Nursing Hamad Medical Corporation Doha Qatar; ^2^ Department of Public Health College of Health Sciences, QU Health Qatar University Doha Qatar; ^3^ Medical Research Center, Hamad Medical Corporation Doha Qatar; ^4^ Department of Community Medicine Rajarajeswari Medical College and Hospital Bengaluru India; ^5^ Department of Nursing, Ibn Sina College for Health Professions Nablus University for Vocational and Technical Education Nablus Palestine; ^6^ Haematology Department King's College Hospital London UK; ^7^ College of Nursing Prince Sattam bin Abdulaziz University Al‐Kharj Saudi Arabia; ^8^ Psychiatric and Mental Health Nursing Department Alexandria University Alexandria Egypt; ^9^ Graduate School Program St. Paul University Tuguegarao Philippines; ^10^ Department of Medicine Services Institute of Medical Sciences Lahore Pakistan; ^11^ Faculty of Pharmacy Zarqa University Zarqa Jordan; ^12^ Faculty of Nursing Cairo University Cairo Egypt; ^13^ Community Health Nursing Department College of Nursing Jouf University Sakaka Saudi Arabia; ^14^ Department of Clinical Pharmacy and Therapeutics School of Pharmacy Applied Science Private University Amman Jordan; ^15^ Nursing Department King Abdullah Medical City Makkah Saudi Arabia; ^16^ College of Health Sciences University of Doha for Science and Technology Doha Qatar; ^17^ Department of Otolaryngology Ambulatory Care Center, Hamad Medical Corporation Doha Qatar; ^18^ Clinical and Pharmacy Practice Department College of Pharmacy, QU Health Qatar University Doha Qatar

**Keywords:** cross‐sectional study, GLP‐1 receptor agonists, healthcare providers, obesity, weight management

## Abstract

**Background and Aim:**

Glucagon‐like peptide‐1 receptor agonists (GLP‐1RAs) were initially developed for type 2 diabetes but have gained widespread use for weight management, including among non‐diabetic individuals. This study aimed to estimate the prevalence of GLP‐1RA use, describe usage patterns, and explore healthcare providers' (HCPs) perceptions of their efficacy and safety.

**Methods:**

A cross‐sectional study was conducted among 657 HCPs from 10 countries using a structured online survey between September and December 2023. Convenience sampling was employed, statistical analyses were performed using STATA 17. Associations between demographic characteristics and perceptions of GLP‐1RAs were analyzed using the chi‐square test.

**Results:**

Among 657 HCPs, 59.2% were female and 40.8% were male, with a median age of 36.0 years (IQR: 29.0–44.0) and a median BMI of 26.7 (IQR: 23.8–30.7). Among professional groups, nurses accounted for 44.7%, followed by physicians (36.2%) and allied health professionals (18.7%). Semaglutide (45.7%, 95% CI: 41.8%−49.5%) was the most commonly used GLP‐1RA, followed by Liraglutide (36.9%, 95% CI: 33.2%−40.8%). Other GLP‐1RAs were used less frequently, including Dulaglutide (17.0%, 95% CI: 14.2%−20.1%), Exenatide (14.1%, 95% CI: 11.5%−17.0%), Albiglutide (7.0%, 95% CI: 5.1%−9.2%), and Lixisenatide (8.5%, 95% CI: 6.5%−10.9%. Regarding perceived safety, 68.6% considered GLP‐1RAs safe. Safety perceptions were significantly associated with educational level (*p* = 0.022), with participants holding higher degrees being more likely to perceive GLP‐1RAs as unsafe (18.3%) compared to those with a bachelor's degree or lower (10.8%). No associations were found with age (*p* = 0.487), sex (*p* = 0.729), or BMI (*p* = 0.089). Similarly, 73.5% of participants considered GLP‐1RAs effective for perceived efficacy. No associations were found with sex (*p* = 0.663) or BMI (*p* = 0.446). Older participants (*p* = 0.011) and those with higher education (*p* = 0.006) were more likely to perceive GLP‐1RAs as ineffective.

**Conclusion:**

This study provides the first prevalence estimate of GLP‐1RA use among HCPs and GLP1‐Ras users and explores the associations between demographic characteristics and perceptions of safety and efficacy. The findings highlight the self‐prescribing practices of these medications for weight management and underscore the need for appropriate monitoring to avoid potential health risks.

## 1 Introduction

The World Health Organization (WHO) reports that every year, at least of 2.8 million people die as a result of complications related to obesity [1]. This ongoing pandemic has been developing insidiously over decades. Evidence suggests that achieving 5%−10% weight loss can significantly mitigate these complications and enhance the quality of life [2].

Glucagon‐like peptide‐1 receptor agonist medications (GLP‐1RAs) have emerged as a non‐invasive approach to achieving substantial weight loss goals, representing a promising avenue for weight management. They are at the forefront of pharmacological interventions for obesity and have been recognized as breakthrough treatments in 2023 [3]. GLP‐RAs include: Semaglutide, Liraglutide, Exenatide, Dulaglutide, Lixisenatide, and Albiglutide [4]. Also, they are used in managing type 2 diabetes (T2DM), particularly in patients with a body mass index (BMI ≥ 30 kg/m^2^. These medications not only help in improving glycemic control but also contribute to significant weight loss, making them a valuable option for individuals with both obesity and diabetes.

Currently, the FDA has approved Semaglutide, Liraglutide, and Tirzepatide for chronic weight management in adults with obesity (BMI ≥ 30 kg/m²) or overweight (BMI ≥ 27 kg/m²) with at least one weight‐related condition such as T2DM, hypertension, or hyperlipidemia.

GLP‐1RAs mimic the effects of a naturally produced hormone, GLP‐1 [5]. which plays a key role in glucose regulation and appetite [5]. These medications slow gastric emptying, prolonging the sensation of satiety post‐meal and diminishing appetite through their effects on the brain's hunger and craving centers [6]. They are effective for both type 2 diabetes management and weight reduction as they improve blood glucose control while reducing calorie intake [7]. Our study targeted HCPs without diabetes who have used GLP‐1RAs solely as a pharmacological strategy for weight management within the last year, and aims to explore three main objectives:

1‐Determine the prevalence of GLP‐1RAs use among non‐diabetic healthcare providers (HCPs), 2‐Identify the patterns of GLP‐1RAs use, and their associations with the HCPs demographic characteristics such as age, sex, education, and BMI categories, 3‐Assess HCPs perceptions regarding the safety and efficacy of GLP‐1 RAs use.

## 2 Methods

### 2.1 Study Design and Participants

This multicounty cross‐sectional study was conducted from September to December 2023. This study was reported following the Strengthening the Reporting of Observational Studies in Epidemiology (STROBE) guidelines [8].

### 2.2 Ethical Approval

The study was approved by the Institutional Review Board (IRB) in Hamad Medical Corporation (HMC) (Ref number: MRC‐01‐23‐315). The study was conducted in accordance with Helsinki's declaration. Informed consent was obtained from the participants before completing the online survey. Throughout the study, confidentiality of participant data was maintained. As for international collaboration, the participating countries typically adopted one of two approaches: they either accepted the IRB approval from the primary research organization, HMC, or they pursued their own IRB approval process through their respective local or national ethical review committees. The IRB has approved the study at the Applied Science Private University, Jordan (2023‐PHA‐34) and the IRB at King Abdullah Medical City, Saudi Arabia (23‐1162).

### 2.3 Study Population and Setting

We recruited participants from diverse HCPs, including nurses, doctors, and allied health workers, from 10 countries (Egypt, India, Iraq, Jordan, Saudi Arabia, Lebanon, Pakistan, Palestine, the Philippines, and Qatar). For participants to be eligible, they must be HCPs who have recently personal use of GLP‐1RAs for weight management only. Those who declined to participate were excluded from the study.

### 2.4 Sampling Strategy and Sample Size Calculation

A convenience sampling strategy was employed. With 657 participants, the study can estimate the prevalence of any type of GLP‐1 among users with a maximum margin of error of ±4% at 95% confidence intervals (CI) based on the Wald Method.

### 2.5 Survey

Face validity was established through iterative expert reviews, ensuring the survey items were clear, relevant, and appropriate for assessing our objectives. It consists of two main parts: Section A, an introduction, and a screening section. This section begins with an introductory question to determine whether the respondent has ever used GLP‐1 RAs for weight management. This screening step ensures that only relevant respondents proceed with the survey. If the participant is eligible, he/she completes the demographics and professional background section. We collected demographic information (age, gender, weight, height, HbA1c levels) and the participants' professional background details (educational level, professional role, practicing country). This data helps analyze the responses in the context of the respondents' backgrounds.

Section B‐ the use of GLP‐1 Receptor agonists. This section delves into the specifics of GLP‐1 RAs use utilizing the Likert scale and covering several areas:

▪ ***Indications***: Participants are asked about the indications for which they have used GLP‐1 receptor agonists, including weight management in non‐diabetic patients or other off‐label use such as Polycystic Ovary Syndrome (PCOS), pre‐diabetes, and others.
▪ ***Doses***: This query determines whether the off‐label dose(s) is below, within, or above the recommended range.
▪ ***Reasons***: Respondents provide their main reasons for using GLP‐1RAs, such as limited treatment options, previous positive experiences, Recommendations from Colleagues/Friends/Relatives, social media advertisements, published literature, and other reasons.
▪ ***Safety, Efficacy, and Knowledge*** **:** This part assesses perceptions of the safety and efficacy of GLP‐1 RAs use, observed adverse events, and whether there are official guidelines or formal education/training.

### 2.6 Data Collection

We leveraged social media platforms like LinkedIn, X (formerly Twitter), and Facebook professional groups to reach and recruit diverse HCPs from multiple countries. This approach involved designing a post communicated the study's objectives, inviting active participation, and guiding interested individuals to a centralized SharePoint link for further information and enrollment.

### 2.7 Statistical Analysis

The statistical analysis was performed using STATA 17 software (College Station, TX: Stata Corp LP). Data was thoroughly checked and cleaned. Descriptive statistics were employed to summarize the data; Categorical variables were expressed as a percentage (%) and frequencies, while skewed continuous data were expressed as median and interquartile range. Inferential statistics were used to estimate the prevalence of GLP‐1Ras use, prevalence was expressed as a percentage (%) with 95% CI. The participants were categorized based on the WHO's BMI index classification [9]. For a more insightful analysis, we merged the overweight and obese categories into a single group, as detailed in Table 1.

**Table 1:** Demographic profile and patterns of GLP‐1 receptor agonists use among HCPs users.

Questions	*N* = 657 (*n*, %).
What is your Age? (Median year, IQR)	36.0 (29.0−44.0)
What is your Age? (categories)	
23−33 years	243 (41.8.0%)
34−44 years	201 (34.5%)
45−55 years	105 (18.0%)
56−66	30 (5.2%)
Above 67	3 (0.5%)
BMI (median, IQR)	26.7 (23.8−30.7)
*BMI*	
Underweight	12 (1.8%)
Normal weight	224 (34.1%)
Overweight and obese	421 (64.1%)
*What is your sex?*	
Female	389 (59.2%)
Male	268 (40.8%)
*What are your HbA1c levels?*	
Normal	404 (61.5%)
Prediabetes	109 (16.6%)
I don't know	85 (12.9%)
Diabetes	59(9.0%)
*For what off‐label indication have you used GLP‐1RAs*	
Weight management in non‐diabetic patients	354 (53.9%)
Wt and Pre‐diabetes	111 (16.9%)
Pre‐diabetes	106 (16.1%)
PCOS	60 (9.1%)
PCOS and Wt management	13 (2.0%)
PCOS and pre‐diabetes	11 (1.7%)
No reason	2 (0.3%)
*What is your highest educational level?*	
Bachelor's degree	324 (49.2%)
Master's degree	252 (38.4%)
Ph.D. degree	71 (10.8%)
Others	10 (1.6%)
*What is your professional role?*	
Nurses	294 (44.75%)
Doctors	238 (36.2%)
Allied health	123 (18.7%)
Others	2 (0.3%)
*In which Country do you practice?*	
Egypt	235 (35.8%)
India	184 (28.0%)
Pakistan	74 (11.3%)
Jordan	65 (9.9%)
Qatar	49 (7.5%)
KSA	14 (2.1%)
Lebanon	16 (2.4%)
Philippines	10 (1.5%)
Iraq	6 (0.9%)
Palestine	3 (0.5%)
*For off‐label use, what dose(s) of GLP‐1 RAs do you use*	
Within the recommended range	404 (61.5%)
Lower than recommended	179 (27%)
Higher than recommended	71 (10.8%)
No	1 (0.2%)
No reason	1 (0.2%)
Don't remember	1 (0%)
*What are your main reasons for using GLP‐1 RAs off‐label?*	
Colleague/Friend/Relative recommendation	242 (36.8%)
Previous positive experience	177 (26.9%)
Limited treatment options	113 (17.2%)
Published literature	68 (10.4%)
Social Media Advertisements	49 (7.5%)
Difficulty losing weight	1 (0.2%)
Doctor recommendation	1 (0.2%)
No	1 (0.2%)
No reason	1 (0.2%)
Personal trainer recommended	1 (0.2%)
Recommended by doctor	1 (0.2%)
Prescription	1 (0.2%)
*Overall, how do you perceive the safety?*	
Somewhat safe	271 (41.2%)
Very safe	180 (27.4%)
Neither safe nor unsafe	111 (16.9%)
Somewhat unsafe	80 (12.2%)
Very unsafe	15 (2.3%)
*Have you observed any adverse events associated with the off‐label use of GLP‐1RAs*	
No	350 (53.3%)
Maybe	188 (28.6%)
Yes	119 (18.1%)
*How do you perceive the efficacy of off‐label use of GLP‐1 RAs?*	
Somewhat effective	305 (46.4%)
Very effective	178 (27.1%)
Neither effective nor ineffective	99 (15.1%)
Somewhat ineffective	65 (9.9%)
Very ineffective	10 (1.5%)
*Are there any official guidelines or recommendations in your Country*	
No	324 (49.3%)
Maybe	168 (25.6%)
Yes	165 (25.1%)
*Have you ever received formal education/training on the off‐label use of GLP‐1 RAs?*	
No	333 (50.7%)
Yes	165 (25.1%)
Maybe	159 (24.2%)

Associations between safety perception and demographic variables, mainly age, sex, education, and BMI categories, were assessed using the Chi‐squared test with Fisher's exact test when expected counts fall below 5. For such analysis, answers to the perception question were recoded into “safe” which included “very safe” and “somewhat safe,” “unsafe” which included “very unsafe” and “somewhat unsafe” and “neither” for those who answered, “neither safe nor unsafe” The same analysis was also done for the efficacy perception. All *p* values reported were two‐sided p‐values. A *p* value that is lower than 0.05 was considered statistically significant.

### 2.8 Role of the Funding Source

This study was funded by the Medical Research Center at HMC (MRC‐01‐23‐315). The funder had no role in the study design, data collection, analysis, interpretation, or report writing.

## 3 Results

### 3.1 Demographic Characteristics of GLP‐1RAs Users

Our study included 657 healthcare professionals (HCPs), with a higher proportion of female participants (59.2%) compared to males (40.8%). The median BMI was 26.7 (IQR: 23.8−30.7)

The overall median age of participants was 36.0 years (IQR: 29.0–44.0). While there was some age diversity, younger participants (23–44 years) constituted the majority (67.5%), followed by middle‐aged individuals (45–55 years) at 16.0%, with only 5.0% aged 56 years or older. the majority of respondents practice in Egypt (35.8%) and India (28.0%), followed by Pakistan (11.3%), Jordan (9.9%), and Qatar (7.5%). Smaller proportions of respondents reported practicing in KSA (2.1%), Lebanon (2.4%), the Philippines (1.5%), Iraq (0.9%), and Palestine (0.5%).

Nurses represented the largest group, 44.7% (*n* = 294), of whom 189 held a bachelor's degree, 76 had a master's degree, and 24 held a Ph.D. Physicians comprised 36.2% (*n* = 238), with 76 holding a bachelor's degree, 140 having a master's degree, and 21 possessing a Ph.D. Allied health professionals accounted for 18.7% (*n* = 123), including 60 with a bachelor's degree, 34 with a master's degree, and 26 with a Ph.D. Further details are presented in Table 1.

### 3.2 Prevalence of Different Types of GLP‐1RAs Use Among HCPs GLP‐1RAs Users

The survey findings presented in Table 2 indicate that Semaglutide was the most frequently used GLP‐1RA, with a prevalence of 45.6% (95% CI: 41.8%–49.5%). Liraglutide was the second most commonly used GLP‐1RA, with a prevalence of 36.9% (95% CI: 33.2%–40.8%).

**Table 2:** Prevalence of GLP‐1 RAs use among 657 HCPs users.

GLP‐1 RAs medication	Prevalence *n* (%)	95% CI
Semaglutide	300 (45.6%)	41.8%−49.5%
Liraglutide	243 (36.9%)	33.2%−40.8%
Dulaglutide	112 (17.0%)	14.2%−20.1%
Exenatide	93 (14.1%)	11.5%−17.0%
Albiglutide	46 (7.0%)	5.1%−9.2%
Lixisenatide	56 (8.5%)	6.5%−10.9%

Dulaglutide and Exenatide were used by 17.0% (95% CI: 14.2%–20.1%) and 14.1% (95% CI: 11.5%–17.0%) of participants, respectively. Lixisenatide had a prevalence of 8.5% (95% CI: 6.5%–10.9%), while Albiglutide had the lowest reported prevalence at 7.0% (95% CI: 5.1%–9.2%).

### 3.3 Perceived Safety and Efficacy

Regarding the perceived safety of GLP‐1Ras usage, our analysis revealed that 68.6% of participants considered these medications to be safe (41.2% somewhat safe, 27.4% very safe), whereas 14.5% perceived them as unsafe. (12.2% somewhat unsafe, 2.3% very unsafe).

Our analysis found no significant difference in perceived safety by age (*p* = 0.487), sex (*p* = 0.729), and by BMI categories (*p* = 0.089). Notably, a significant statistical association exists between safety perceptions and educational level (*p* = 0.022). The percentage of participants who perceived the off‐label use of GLP1‐RAs as less safe is significantly higher among participants with more than bachelor's degrees as compared to those with bachelor's degrees or lower (18.3% vs. 10.8%, *p* = 0.022), Further details are presented in Table 3.

**Table 3:** Association between perceived safety and demographic characteristics of HCPs.

Demographics	Safe (*n*, %)	Neither (*n*, %)	Not Safe (*n*, %)	*p* value
*Age group*				0.487
23–33 years	164 (67.5%)	39 (16.0%)	40 (16.5%)	
34–44 years	143 (71.1%)	38 (18.9%)	20 (10.0%)	
45–55 years	73 (69.5%)	13 (12.4%)	19 (18.1%)	
56 years and above	21 (63.6%)	6 (18.2%)	6 (18.2%)	
*Sex*				0.729
Female	266 (68.4%)	69 (17.7%)	54 (13.9%)	
Male	185 (69.0%)	42 (15.7%)	41 (15.3%)	
*Education*				0.022*
Bachelor's degree or below	237 (71.0%)	61 (18.3%)	36 (10.8%)	
Above bachelor's degree	214 (66.3%)	50 (15.5%)	59 (18.3%)	
*BMI category*				0.089
Underweight	9 (75.0%)	2 (16.7%)	1 (8.3%)	
Normal	160 (71.4%)	36 (16.1%)	28 (12.5%)	
Overweight and Obese	282 (67.0%)	73 (17.3%)	66 (15.7%)	

*Note: p* values are based on chi‐square tests. Statistically significant associations

(*p* < 0.05) are indicated with an asterisk*.

Regarding the perceived efficacy of GLP‐1RA use, 73.5% of participants considered these medications to be effective (46.4% somewhat effective, 27.1% very effective), whereas 11.4% perceived them as ineffective (9.9% somewhat ineffective, 1.5% very ineffective).

The percentage of HCPs who perceived GLP‐1RAs as effective was significantly lower among those aged 56 years and above compared to younger age groups (48.5% vs. 72.0%–79.1%, *p* = 0.011*) and significantly lower among HCPs with post graduate degree as compared to HCPs with bachelor's degrees or lower (70.0% vs. 76.9%, *p* = 0.006*), Our analysis found no significant associations between participants perception of efficacy and sex (*p* = 0.663) or BMI categories (*p* = 0.446), Further details are presented in Table 4.

**Table 4:** Association between perceived effectiveness and demographic characteristics of HCPs.

Demographic Variable	Effective (*n*, %)	Neither (*n*, %)	Ineffective (*n*, %)	*p* value
Age Group				0.011*
23–33 years	175 (72.0%)	41 (16.9%)	27 (11.1%)	
34–44 years	159 (79.1%)	26 (12.9%)	16 (8.0%)	
45–55 years	78 (74.3%)	12 (11.4%)	15 (14.3%)	
56 years and above	16 (48.5%)	9 (27.3%)	8 (24.2%)	
*Sex*				0.663
Female	291 (74.8%)	56 (14.4%)	42 (10.8%)	
Male	192 (71.6%)	43 (16.0%)	33 (12.3%)	
*Education*				0.006*
Bachelor's degree or below	257 (76.9%)	52 (15.6%)	25 (7.5%)	
Above bachelor's degree	226 (70.0%)	47 (14.6%)	50 (15.5%)	
*BMI category*				0.446
Underweight	9 (75.0%)	1 (8.3%)	2 (16.7%)	
Normal	157 (70.1%)	41 (18.3%)	26 (11.6%)	
Overweight and Obese	317 (75.3%)	57 (13.5%)	47 (11.2%)	

*Note: p* values are based on chi‐square tests. Statistically significant associations

(*p* < 0.05) are indicated with an asterisk*.

## 4 Discussion

This study was the first to estimate the prevalence and explore the association between the HCPs characteristics and their perceptions of the efficacy and safety of GLP‐1Ras.

### 4.1 Demographics and GLP‐1RA Usage Patterns

41% of participants using GLP‐1RAs were male, reflecting a growing trend of body dissatisfaction among men. This challenges the widely accepted notion that women experience greater body image dissatisfaction due to societal norms and idealized body standards [10].

Emerging evidence suggests that while men often report lower body dissatisfaction in surveys, this may be due to measurement tools predominantly assessing fat‐related concerns, which are traditionally emphasized among women [11]. Instead, male body dissatisfaction is often centered around attaining a leaner, more muscular physique rather than simply weight reduction, diverging from the thin ideal typically associated with female body image concerns.

This does not imply that men are indifferent to their body weight. Instead, their dissatisfaction tends to be more about attaining an idealized form of masculinity, which emphasizes muscularity over simply losing weight.

The majority of participants were young (23–44 years, 68%), which aligns with the growing trend of weight management interest among younger HCPs. Notably, age played a role in the indication for GLP‐1RA use. Younger participants primarily used GLP‐1RAs for weight management (54%), while older HCPs prioritized their use for metabolic conditions such as PCOS and prediabetes. This aligns with existing research indicating that younger individuals are more influenced by aesthetic motivations, whereas older individuals prioritize metabolic health improvements. Moreover, compared to older women, men generally maintain higher levels of self‐esteem in their later years [12].

### 4.2 Prevalence of Different Types of GLP‐1RAs Use Among Hcps GLP‐1RAs Users

Our findings indicate a high prevalence of Semaglutide use (46%), which aligns with existing literature [13]. despite its high cost compared to other GLP‐1RAs, one potential driver for high usage could be the availability of Semaglutide in both injectable and oral forms [14, 15], This dual formulation provides convenience for those who prefer oral administration while still accommodating those who opt for the faster onset of action associated with injections. Additionally, practical consideration in dosing may contribute to its high use. Research has shown that once‐weekly dosing of injectable GLP‐1 RAs is associated with better adherence compared to once‐daily dosing, with an 11% lower risk of non‐adherence [15, 16].

Moreover, recent RCTs from the STEP program have consistently demonstrated Semaglutide superiority in promoting weight loss over Liraglutide [17]. On average, Semaglutide users experienced an 11%–14% reduction in body weight, while those using Liraglutide had a 7.8% reduction [17, 18, 19, 20, 21, 22, 23, 24, 25, 26, 27, 28, 29, 30, 31, 32, 33, 34]. Additionally, economic evaluations suggest that Semaglutide 2.4 mg is cost‐effective, with a willingness‐to‐pay (WTP) threshold of CAD 50,000 per quality‐adjusted life year (QALY). A QALY is a measure used in health economics to assess the value of medical interventions, incorporating both quality and quantity of life gained. The CAD 50,000 per QALY threshold is widely used in economic evaluations as a benchmark for cost‐effectiveness, indicating that if a treatment provides a year of perfect health for less than this amount, it is considered a good investment for healthcare systems. This reinforces the economic justification for Semaglutide's use, particularly given its superior weight loss efficacy, metabolic benefits, and the potential to reduce obesity‐related complications, which can significantly lower long‐term healthcare costs [35].

Liraglutide was the second most prevalent GLP‐1RA (36.9%) is consistent with its longer market presence and established safety profile. The LEADER trial demonstrated cardiovascular benefits of liraglutide, showing a 13% reduction in major adverse cardiovascular events compared to placebo in patients with type 2 diabetes and high cardiovascular risk [36]. The lower prevalence rates of dulaglutide (17.0%), exenatide (14.1%), lixisenatide (8.5%), and albiglutide (7.0%) may reflect their comparative efficacy profiles or administration considerations. For instance, the once‐weekly administration of dulaglutide offers convenience advantages, but its weight loss effects are generally less pronounced than those of semaglutide.

The preference patterns observed among HCPs to use mostly semaglutide, liraglutide, or dulaglutide may also reflect the influence of recent cardiovascular outcome trials [29, 36, 37] for GLP‐1 RAs with proven cardiovascular benefits [28, 35, 36]. suggesting that cardioprotective effects may be a key consideration in their selection by HCPs.

### 4.3 Perceived Safety and Efficacy

Our results indicate that HCPs generally hold favorable views toward GLP‐1RAs, with 68.6% considering these medications safe and 73.5% perceiving them as effective. These positive perceptions align with the substantial body of evidence from clinical trials and real‐world studies supporting both the safety profile and therapeutic benefits of GLP‐1Ras [15, 17, 21, 22, 23, 24, 25, 38].

Education level emerged as a significant factor influencing both safety and efficacy perceptions among HCPs. Those with postgraduate qualifications expressed greater concerns about both safety and efficacy compared to those with lower educational attainment. This pattern suggests that advanced education fosters a more critical evaluation of medication benefits and risks, potentially due to greater exposure to scientific literature discussing clinical trial limitations and emerging safety signals. Higher educational levels may enhance critical appraisal skills, leading to more cautious perceptions of medication safety and efficacy. While the direct relationship between academic degree level and perceptions of medication safety and efficacy requires further investigation, our results suggests that educational background significantly influences HCPs' clinical perceptions, confidence, and practices in ways that likely extend to medication‐related judgments [39].

Age was significantly associated with efficacy perceptions (*p* = 0.011) but not safety perceptions (*p* = 0.487). HCPs aged 56 years and above were notably less likely to perceive GLP‐1RAs as effective (48.5%) compared to younger age groups (72.0%−79.1%).

Understanding physician perceptions of GLP‐1RAs is indeed important, as significant discrepancies exist between clinical evidence and how HCPs view these medications. For example, physicians reported an average patient weight loss of 9.22% with GLP‐1RAs, which is substantially lower than the 14.9% and 18.5% reported in the STEP and SURMOUNT clinical trials. Similarly, physicians estimated side effect rates at 32.62%, markedly lower than trial‐reported rates of 80%−90% [40].

These perception gaps extend to understanding cardiovascular benefits as well. Only 48.4% of physicians recognized cardiovascular benefits in diabetic patients (consistent with random guessing), and just 39.3% recognized these benefits in non‐diabetic patients. This suggests a significant knowledge deficit regarding the evidence‐based benefits of these medications [40].

Rather than focusing on researching off‐label use itself, which is indeed common practice among HCPs, research should address these gaps in perceptions. The discrepancy between physician perceptions and clinical evidence could impact patient care, medication adherence, and clinical outcomes. Physicians who underestimate efficacy might be less likely to prescribe these medications or may not provide patients with realistic expectations, while underestimating side effects could leave patients unprepared for common adverse events.

### 4.4 Clinical and Policy Implications

this study highlights the growing issue of obesity among HCPs [41], while HCPs have the medical knowledge to self‐prescribe, it is generally recommended that they seek independent medical advice to ensure objective assessment and monitoring. Self‐prescribing, especially for off‐label uses such as weight management, can lead to ethical dilemmas and potential health risks [42]. Therefore, HCPs considering GLP‐1 receptor agonists for personal use should consult with a qualified colleague to discuss the benefits, risks, and appropriate monitoring associated with these medications. Addressing obesity within the healthcare workforce through comprehensive wellness programs and alternative interventions could help reduce reliance on pharmacologic solutions.

Our findings suggest that HCPs are not immune to the same societal pressures influencing the general population regarding body image and weight management [43]. The observed divergence between generally positive perceptions (approximately 70% favorable for both safety and efficacy) and the more cautious views held by specific demographic subgroups (particularly those with advanced education and older groups) warrants further investigation. This pattern could indicate potential knowledge gaps or differing interpretations of available evidence that might influence clinical decision‐making and patient counseling regarding GLP‐1RA therapy. Additionally [39].

## 5 Limitations

While this study offers important insights into the use of GLP‐1RAs for weight management among HCPs, it is essential to interpret the findings in light of its limitations. Specifically, the study's reliance on self‐reported data via a structured questionnaire may introduce response bias. Participants might overestimate or underestimate their usage patterns, reasons for off‐label use, and perceptions of efficacy and safety. This reliance on self‐reporting questions the precision and trustworthiness of the collected data, which could impact the validity of the study's conclusions. Convenience sampling facilitated recruitment but introduced selection bias; additionally, the predominance of data from Egypt and India limits generalizability to other regions. Another limitation arises from the recruitment modalities, particularly the reliance on social media platforms. This approach may introduce an additional layer of sampling bias, as the population accessible through social media may not represent the general HCP community. Social media users' demographic and professional characteristics could differ significantly from those not active on these platforms, which might influence the study results. Despite these limitations, this study provides preliminary insights into an emerging trend of GLP‐1RA use among HCPs. The findings highlight different types of use and the relationship between uses perception of safety and efficacy with demographic variables. Also, the study included a diverse group of HCPs from different countries, different age groups, both sexes, different educational levels. This enriches the generalizability of the study results to similar HCPs. We acknowledge that this is a descriptive study and there is the need for further research using more rigorous methodologies, probability‐based sampling, and longitudinal designs, to generate more information; Comprehensive wellness program should be created and tested as a good percentage of HCPs consider the off‐label use as safe and efficacious. Additionally, as the accessibility of these medications expands through online and telehealth platforms, future research should explore the regulatory and ethical considerations surrounding their use, ensuring patient safety and responsible prescribing practices.

## 6 Conclusion

Our study reveals that HCPs increasingly use GLP‐1 receptor agonists for weight management beyond approved indications, with semaglutide being the most commonly used medication. We identified important perceptions gaps regarding safety and efficacy profiles of these medications. These perception discrepancies could influence both personal use patterns and patient care decisions. Our findings highlight the need for comprehensive education programs and clear guidelines for off‐label use to improve safety monitoring and optimize clinical outcomes.

## Author Contributions

**Abdulqadir J. Nashwan:** conceptualization, writing – original draft, writing – review and editing, data curation, methodology, supervision. **Hana J. Abukhadijah:** writing – original draft, writing – review and editing, data curation, formal analysis. **Vidusha Karavadi:** writing – original draft, writing – review and editing, data curation. **Ibrahim Aqtam:** writing – original draft, writing – review and editing, data curation. **Anas Ibraheem:** writing – original draft, writing – review and editing, data curation. **Prakash Palanivelu:** writing – original draft, writing – review and editing, data curation. **Mahmoud A. Khedr:** writing – original draft, writing – review and editing, data curation. **Abdulkarim O. Agga:** writing – original draft, writing – review and editing, data curation. **Obaid Ur Rehman:** Writing – original draft, Writing – review and editing, Data curation. **Eeshal Fatima:** writing – original draft, writing – review and editing, data curation. **Mohammad A. Abu Asal:** writing – original draft, writing – review and editing, data curation. **Rana Abutaima:** writing – original draft, writing – review and editing, data curation. **Marwa M. Shaban:** writing – original draft, writing – review and editing, data curation. **Mostafa Shaban:** writing – original draft, writing – review and editing, data curation. **Muna Barakat:** writing – original draft, writing – review and editing, data curation. **Nasser M. Aldosari:** writing –original draft, writing – review and editing, data curation. **Albara M. Alomari:** writing – original draft, writing – review and editing, data curation. **Adham A. Aljariri:** writing – original draft, data curation, writing – review and editing. **Nabeel F. Al‐Lobaney:** writing – original draft, writing – review and editing, data curation. **Mutaz I. Othman:** writing – original draft, writing – review and editing, data curation. **Ahmad A. Abujaber:** writing – original draft, writing – review and editing, formal analysis, data curation. **Kholoud Bastaki:** writing – original draft, writing – review and editing.

## Conflicts of Interest

The authors declare no conflict of interest. Abdulqadir J. Nashwan is an Editorial Board member of Health Science Reports and a co‐author of this article. To minimize bias, he was excluded from all editorial decision‐making related to the acceptance of this article for publication. The other authors declare no conflicts of interest.

## Transparency Statement

The lead author Abdulqadir J. Nashwan affirms that this manuscript is an honest, accurate, and transparent account of the study being reported; that no important aspects of the study have been omitted; and that any discrepancies from the study as planned (and, if relevant, registered) have been explained.

## Supporting information

STROBE checklist.

## Data Availability

The data are available from the corresponding author on reasonable request.
